# The effects of ruminant milk treatments on hippocampal, striatal, and prefrontal cortex gene expression in pigs as a model for the human infant

**DOI:** 10.3389/fnins.2022.937845

**Published:** 2022-08-15

**Authors:** Ankita Jena, Carlos A. Montoya, Wayne Young, Jane A. Mullaney, Debashree Roy, Ryan N. Dilger, Caroline Giezenaar, Warren C. McNabb, Nicole C. Roy

**Affiliations:** ^1^Riddet Institute, Massey University, Palmerston North, New Zealand; ^2^School of Food and Advanced Technology, College of Sciences, Massey University, Palmerston North, New Zealand; ^3^AgResearch, Palmerston North, New Zealand; ^4^High-Value Nutrition National Science Challenge, Auckland, New Zealand; ^5^Department of Animal Sciences, University of Illinois, Urbana, IL, United States; ^6^Food Experience and Sensory Testing (FEAST) Laboratory, School of Food and Advanced Technology, Massey University, Palmerston North, New Zealand; ^7^Department of Human Nutrition, University of Otago, Dunedin, New Zealand

**Keywords:** nutrition, early life, cognition, pig, gut-brain axis, brain gene expression, ruminant milk

## Abstract

While infant formula is usually bovine milk-based, interest in other ruminant milk-based formulas is growing. However, whether different ruminant milk treatments with varying nutrient compositions influence the infant’s brain development remains unknown. The aim was to determine the effects of consuming bovine, caprine, or ovine milk on brain gene expression in the early postnatal period using a pig model of the human infant. Starting at postnatal day 7 or 8, pigs were exclusively fed bovine, ovine, or caprine milk for 15 days. The mRNA abundance of 77 genes in the prefrontal cortex, hippocampus, and striatum regions was measured at postnatal day 21 or 22 using NanoString. The expression level of two hippocampal and nine striatal genes was most affected by milk treatments, particularly ovine milk. These modulatory genes are involved in glutamate, gamma-aminobutyric acid, serotonin, adrenaline and neurotrophin signaling and the synaptic vesicle cycle. The expression level of genes involved in gamma-aminobutyric acid signaling was associated with pigs’ lactose intake. In contrast, milk treatments did not affect the mRNA abundance of the genes in the prefrontal cortex. This study provides the first evidence of the association of different ruminant milk treatments with brain gene expression related to cognitive function in the first 3 months of postnatal life.

## Introduction

The early postnatal years of life are critical in determining developmental, behavioral, and health outcomes in later life. Rapid synaptogenesis, myelination, and the establishment of cognitive abilities mark developmental changes in this period. In addition, genetic [e.g., brain-derived neurotrophic factor (BDNF), neuregulin] and environmental (e.g., nutrition, prenatal care) factors influence the development of the brain (Valadares et al., 2010; Knickmeyer et al., 2014). Genetic factors dictate the *in utero* brain development stages, but environmental factors mainly influence postnatal brain development. Hence, any environmental insult or stimuli during this period could affect the brain performance in adulthood.

Human breast milk is the optimal source of nutrition for infants, but infant formula is an alternative or complementary solution in situations where human breast milk is unavailable or limited. Milk, regardless of the species it comes from, contains different lipids (e.g., phospholipids, sphingomyelin, polyunsaturated fatty acids), proteins (e.g., lactoferrin), carbohydrates (e.g., lactose, oligosaccharides), vitamins and minerals (e.g., vitamin B, choline, iron), with many of these nutrient sources increasingly recognized for their associations or roles in brain development postnatally (Lin et al., 2019).

A study by Deoni et al. (2018) showed that a formula containing a high amount (62 mg/L) of sphingomyelin increases brain myelination and improves cognitive performance in infants compared with a low amount (28 mg/L) of sphingomyelin. Another study reported an association between the concentration of oligosaccharide 2-fucosyllactose in breast milk at 1 month of lactation and improved cognitive function in infants aged 24 months (Berger et al., 2020). In addition, inadequate protein intake during early postnatal life was associated with learning and memory impairments (Valadares et al., 2010), reduced brain weight, and dendritic arborization (Chertoff, 2015) in rodents.

The effects of early postnatal nutrition on brain development have been studied primarily in the context of behavior, but limited studies have focused on the underlying changes in molecular features in the brain tissue (Fleming et al., 2020; Page and Anday, 2020). For instance, a study showed that feeding human or bovine milk oligosaccharides from postnatal day (PND) 2–32 to pigs either increases or decreases the hippocampal expression of neurotransmitter receptor (*GABRB2*, *GLRA4*, and *CHRM3*) and transporter (*SLC1A7*) genes and improves recognition memory, suggesting a link between nutrient-gene-behavior (Fleming et al., 2020). Hence, more studies are essential to better understand the effect of nutrition on brain gene expression that may act as a determinant of cognitive abilities and behavior in early postnatal life.

Bovine milk is the most common ruminant milk used for infant formula (Martin et al., 2016). However, caregivers have increased interest in using formula made with other ruminant milk, including ovine and caprine sources. This expansion in the use of non-bovine milk is primarily due to the association of bovine milk consumption with the development of allergies in infants and reduced symptoms with consumption of ovine or caprine milk (Park, 1994; Masoodi and Shafi, 2010). Ovine and caprine milk also have a greater nutritional value than bovine milk for specific nutrients. Ovine milk contains higher protein, lipids, vitamins (riboflavin and vitamin C), minerals (calcium and phosphorous), and energy (Park et al., 2007; Barlowska et al., 2011; Claeys et al., 2014), while caprine milk has more oligosaccharides and a profile of oligosaccharides closer to that of human breast milk (Viverge et al., 1997; Martinez-Ferez et al., 2006).

Previous *in vitro* (Roy et al., 2021) and *in vivo* (Roy et al., 2022) studies have shown that gastric digestion and stomach emptying rate of nutrients differed across ruminant milk. These differences would likely affect the availability of nutrients for small intestinal absorption, fermentation by the resident microbiota in the gut, tissue metabolism, and brain function. Another *in vitro* study showed that bovine and ovine milk fermentation using infant fecal inoculum resulted in differences in the relative abundance of the microbiota and their metabolites between milk types (Ahlborn et al., 2020). In addition, microbiota and microbial metabolites are increasingly recognized for their potential to influence brain function by participating in the gut-brain axis (O’Mahony et al., 2015; Gao et al., 2019; Kelsey et al., 2021; Tamana et al., 2021). However, despite these differences between ruminant milk, their effects on the activity of the different brain regions with specific roles in cognitive development (e.g., the hippocampus, striatum, and prefrontal cortex) (Lavenex and Banta Lavenex, 2013; Wierenga et al., 2014; Werchan et al., 2016), are ill-defined.

The effects of bovine, ovine, and caprine milk on the gene expression of the prefrontal cortex, hippocampus, and striatum were determined from samples collected as part of a study focusing on structural changes in bovine and non-bovine (ovine and caprine) whole milk on digestion of pigs in early postnatal life published elsewhere (Roy et al., 2022). The hypothesis of this secondary analysis was that differences in nutrient composition between the three main ruminant milk used to make infant formula led to differences in gene expression of the brain areas associated with cognitive function.

## Materials and methods

### Milk chemical composition analysis

Raw whole milk batches from bovine, caprine and ovine species were obtained under chilled conditions from the Massey University No. 4 Dairy Farm (Palmerston North, New Zealand), Dairy Goat Co-operative (Hamilton, New Zealand) and Phoenix Goats (Palmerston North, New Zealand), and Neer Enterprises Limited (Carterton, New Zealand), respectively. Spray-dried whole milk powders of bovine, caprine, and ovine species were purchased from Davis Food Ingredients, Dairy Goat Co-operative, and Spring Sheep Milk Co., respectively. Premix of vitamin and mineral was purchased from Nutritech International Ltd.

Dry matter was analyzed using an air oven-drying method 990.19, proteins using the Dumas method 968.06, and fats using the Mojonnier method 989.05, respectively. In addition, milk lactose content was measured using a spectrophotometric enzymatic kit (catalog no.-10176303035) (R-Biopharm AG, Germany), and gross energy content was measured using a LECO AC500 bomb calorimeter (LECO Corporation, St. Joseph, MO, United States).

### Experimental animals

This study was reviewed and approved by the Massey University, Animal Ethics Committee (MUAEC protocol 18/97) and described in Roy et al. (2022). Briefly, 24 male pigs at PND 7–8, mean body weight of 3 kg (range 2–3.5 kg) who had consumed *ad libitum* sow’s milk from PND 1 to PND 6 or 7 were received from a commercial farm (Aorere Farms Partnership, Whanganui, New Zealand). Pigs were used as a model because of their comparable nutritional requirements, similar brain developmental patterns, gastrointestinal tract physiology and metabolism to human infants (Moughan et al., 1992; Guilloteau et al., 2010; Mudd and Dilger, 2017). Pigs were housed individually in plastic metabolism crates (700 mm × 450 mm × 500 mm) and each crate was provided with clean toys (which were changed daily) in a room maintained at 28 ± 2°C and under a 16 h light-8 h dark cycle. Under supervision, pigs interacted for 1 h to provide social contact. Technicians and researchers interacted with all the pigs several times daily.

Pigs were randomly allocated to diets at their arrival and were bottle-fed for 15 days exclusively with bovine, caprine or ovine whole milk treatment. The study duration of 15 days was selected as a 3-week-old pig (∼PND 21) has similar developmental patterns to a 3-month-old human (Moughan et al., 1992; Guilloteau et al., 2010). The health of the pigs was monitored throughout the study. Changes in the body temperature (∼38–40°C), defecation frequency, fecal consistency as well as any adverse signs such as dehydration (concentrated urine, skin tenting, and constipation) and scouring were recorded. Milks were well tolerated by the pigs, and no adverse effect on pig’s well-being was observed.

The body weight (BW) of the pigs was recorded on arrival and then every 2 days, and the data were used to adjust the amount of diet offered. From PND 7 or 8 to PND 12 or 13, the pigs were acclimatized and trained to drink from a bottle with a rubber teat. From PND 7 or 8 to PND 18 or 19, pigs were fed a reconstituted whole milk powder diet (including vitamin and mineral supplements). From PND 14 or 15, the diets were balanced for protein content (2 g per kg BW) so that all the pigs from all three diet groups received equal amounts of protein. As protein is one of the main components that influence curd formation in the stomach, matching the protein content allowed the investigation of differences in the rate of whole milk digestion based on milk structure, which was the study’s primary aim (Roy et al., 2022). From PND 19 or 20, a fresh whole milk diet was fed due to a limited supply of fresh ovine and caprine milk. On the last experimental day, fasted (18 h) pigs of PND 21 to 22 were euthanized at 210 min post-feeding to allow the time for nutrients delivery to distant organs. An illustration of the study timeline is given in Supplementary Figure 1.

### Brain tissue sampling

The pigs were anesthetized using a Zoletil 100 (zolazepam and tiletamine, both 50 mg/mL, Zoetis Inc., Parsippany-Troy Hills, NJ, United States) reconstituted with 2.5 mL each of ketamine and xylazine (both 100 mg/mL). The solution containing 50 mg/mL of each drug was administered at a dose rate of 0.4 mL/10 kg of BW by intramuscular injection, followed by euthanization using a lethal dose (0.3 mL/kg BW) of pentobarbitone (Pentobarb 300, Provet NZ Pty Limited). The brain was carefully removed from the skull and immediately dissected on an ice-chilled surface, and the prefrontal cortex, hippocampus, and striatum were collected from the left hemisphere only for consistency with other studies (Zhang et al., 2016; Negi and Guda, 2017). The dissected brain regions were snap-frozen in liquid nitrogen and stored in a −80°C freezer. Brain tissue samples were collected only from 23 pigs as one pig assigned to the caprine milk group inadvertently received another milk.

### RNA extraction

Each brain tissue sample (10–20 mg) was homogenized using a handheld homogenizer for 60 s in 1 mL of QIAzol lysis reagent (Qiagen, Hilden, Germany). The total RNA was extracted using an RNeasy lipid tissue mini kit following the manufacturer’s instructions (Qiagen, Hilden, Germany) and dissolved in 50 μL of RNAase free water. The concentration and the quality of the extracted RNA were evaluated using a NanoDrop^®^ ND-1000 spectrophotometer (Thermo Fisher Scientific, Waltham, MA, United States) and the Agilent 2100 Bioanalyser (Agilent Technologies, Santa Clara, CA, United States), respectively. Samples with an RNA integrity number greater than six were used for gene expression analysis, and this criterion was satisfied by all the brain tissue samples.

### Gene panel selection

A customized panel of 150 genes was created using a literature search and pre-existing NanoString human gene panel for learning, memory, and neurotransmitters. Then, the curation of genes was carried out based on protein existence and entry status in Uniprot. The genes whose evidence was available at transcript and protein levels and whose entry status was reviewed were selected, making a panel of 77 genes (Table 1). The panel consisted of genes related to the brain cellular process (synaptogenesis, myelination, neurotrophins, and synaptic vesicle cycle), neurotransmission (neurotransmitter receptor, transporter, and enzymes involved in neurotransmitter synthesis).

**TABLE 1:** Full NanoString list of genes associated with brain cellular processes and neurotransmission.

Gene symbol	Gene name
*ACHE*	Acetylcholinesterase
*ADRA1D*	Adrenoceptor alpha 1D
*ADRA2A*	Adrenoceptor alpha 2A
*ADRA2B*	Adrenoceptor alpha 2B
*ADRB1*	Adrenoceptor beta 1
*ADRB2*	Adrenoceptor beta 2
*ADRB3*	Adrenoceptor beta 3
*AHR*	Aryl hydrocarbon receptor
*BDNF*	Brain-derived neurotrophic factor
*CHRM1*	Cholinergic receptor muscarinic 1
*CHRM2*	Cholinergic receptor muscarinic 2
*CHRM3*	Cholinergic receptor muscarinic 3
*CHRNA7*	Cholinergic receptor nicotinic alpha 7 subunit
*CHRNB2*	Cholinergic receptor nicotinic beta 2 subunit
*CNP*	2′,3′-Cyclic nucleotide 3′ phosphodiesterase
*CPLX1*	Complexin 1
*CPLX3*	Complexin 3
*CPLX4*	Complexin 4
*DBH*	Dopamine beta-hydroxylase
*DDC*	Dopa decarboxylase
*DLG4*	Disks large MAGUK scaffold protein 4
*DRD1*	Dopamine receptor D1
*DRD2*	Dopamine receptor D2
*GABBR1*	Gamma-aminobutyric acid type B receptor subunit 1
*GABRA1*	Gamma-aminobutyric acid type A receptor subunit alpha 1
*GABRB2*	Gamma-aminobutyric acid type A receptor subunit beta 2
*GABRB3*	Gamma-aminobutyric acid type A receptor subunit beta 3
*GABRG2*	Gamma-aminobutyric acid type A receptor subunit gamma 2
*GAD1*	Glutamate decarboxylase 1
*GAD2*	Glutamate decarboxylase 2
*GAP43*	Growth associated protein 43
*GLS*	Glutaminase
*GRIA2*	Glutamate ionotropic receptor AMPA type subunit 2
*GRID1*	Glutamate ionotropic receptor delta type subunit 1
*GRID2*	Glutamate ionotropic receptor delta type subunit 2
*GRM1*	Glutamate metabotropic receptor 1
*GRM2*	Glutamate metabotropic receptor 2
*GRM6*	Glutamate metabotropic receptor 6
*GRM7*	Glutamate metabotropic receptor 7
*GRM8*	Glutamate metabotropic receptor 8
*HRH1*	Histamine receptor H1
*HTR1B*	5-Hydroxytryptamine receptor 1B
*HTR1D*	5-Hydroxytryptamine receptor 1D
*HTR1E*	5-Hydroxytryptamine receptor 1E
*HTR2A*	5-Hydroxytryptamine receptor 2A
*HTR2B*	5-Hydroxytryptamine receptor 2B
*HTR2C*	5-Hydroxytryptamine receptor 2C
*HTR4*	5-Hydroxytryptamine receptor 4
*MAG*	Myelin associated glycoprotein
*MBP*	Myelin basic protein
*NPY1R*	Neuropeptide Y receptor Y1
*NPY2R*	Neuropeptide Y receptor Y2
*NPY5R*	Neuropeptide Y receptor Y5
*NTF3*	Neurotrophin 3
*NTRK3*	Neurotrophic receptor tyrosine kinase 3
*PLP1*	Proteolipid protein 1
*RAB3A*	Ras-related protein Rab-3A
*RIMS1*	Regulating synaptic membrane exocytosis 1
*SLC1A1*	Solute carrier family 1 member 1
*SLC1A2*	Solute carrier family 1 member 2
*SLC1A3*	Solute carrier family 1 member 3
*SLC22A1*	Solute carrier family 22 member 1
*SLC22A2*	Solute carrier family 22 member 2
*SLC22A3*	Solute carrier family 22 member 3
*SLC5A7*	Solute carrier family 5 member 7
*SLC6A1*	Solute carrier family 6 member 1
*SLC6A11*	Solute carrier family 6 member 11
*SLC6A13*	Solute carrier family 6 member 13
*SNAP25*	Synaptosome associated protein 25
*STX1B*	Syntaxin 1B
*STX3*	Syntaxin 3
*STXBP1*	Syntaxin binding protein 1
*SYN1*	Synapsin I
*SYN2*	Synapsin II
*SYN3*	Synapsin III
*SYT1*	Synaptotagmin 1
*VAMP2*	Vesicle associated membrane protein 2

### Gene expression analysis

Seventy-seven genes were detected using the NanoString nCounter™ system (NanoString Technologies, Seattle, WA, United States). The starting amount of RNA was 300 ng. Each RNA sample of 7 μL was mixed with color-coded capture and reporter probe provided by NanoString. The samples and probes were hybridized at 67°C for 22 h. After hybridization, samples were run on the NanoString nCounter prep station, removing excess probes and immobilizing the sample-probe complex on a cartridge. A nCounter digital analyzer counted the immobilized color-coded complex on the surface of the cartridge.

### Data processing

The raw data (reporter code count files) generated by the analyzer was uploaded into nSolver software (Version 4.0, NanoString Technologies, Seattle, WA, United States). Quality control checks were performed on the raw data using nSolver’s default settings. First, a background correction was carried out by subtracting the number of counts of the highest negative control (out of six negative internal controls provided by NanoString) plus two standard deviations from all the mRNA counts. This step was followed by normalizing individual mRNA counts against the geometric mean of six NanoString positive internal control oligonucleotides and seven reference genes: actin-beta (*ACTB*), beta-2-microglobulin (*B2M*), hypoxanthine phosphoribosyltransferase 1 (*HPRT1*), lactate dehydrogenase A (*LDHA*), phosphoglycerate kinase 1 (*PGK1*), peptidylprolyl isomerase A (*PPIA*), and ribosomal protein L4 (*RPL4*).

### Statistical analyses

All statistical analyses were performed using R (version 4.02). Normalized mRNA count data were log2-transformed prior to statistical analysis. Principal component analysis was performed to compare overall gene expression profiles between treatments. One hippocampal tissue sample of the ovine milk group was identified as an outlier using the criteria of >3 standard deviations from the mean and Hotelling T2 plot (Supplementary Figure 2) and was removed from the study. Differences in expression levels of individual genes between treatment groups and brain regions were analyzed by one-way analysis of variance using the rstatix R package. Brain tissues are different from each other in terms of structure and function. Thus, a one-way analysis of variance for each tissue was conducted to compare the effect of milk treatments. A false discovery rate (FDR) correction was used to reduce the risk of false positives. Genes with FDR < 0.05 or < 0.1 were considered significantly different. The Fisher’s least significant difference test was used for *post hoc* analysis, performed using agricolae package of R. To visualize the significant pairwise differential expression of genes between milk treatment *via* volcano plot, log_2_ fold change (FC) > ±0.5849 (equivalent to FC > 1.5) and FDR < 0.05 (identified using *t*-test) were used. Differential gene expression between brain regions was visualized using the pheatmap R package.

The association between nutrient intake (per kg BW) and brain gene expression was assessed using Spearman correlations. Milk nutrient intake was calculated from PND 9 or 10 onward (Table 2), as the first 3 days of the acclimatization phase had a substantial amount of milk spills and refusals. Spearman rank correlation coefficient and the corresponding *P*-value were calculated using the cor.test function and visualized using the corrplot package in R. Correlations with *P* < 0.05 and rho > ±0.5 were considered significant.

**TABLE 2:** Amounts (g/kg of BW per pig) of bovine, caprine, and ovine whole milk ingested per day.

Component	Bovine	Caprine	Ovine
Dry matter	38.59	35.95	38.46
Protein	10.66	10.15	13.72
Fat	11.96	10.34	13.79
Lactose	13.43	12.55	9.11
Gross energy	223.30	195.58	234.89

## Results

### Overview of gene expression

**FIGURE 1:**
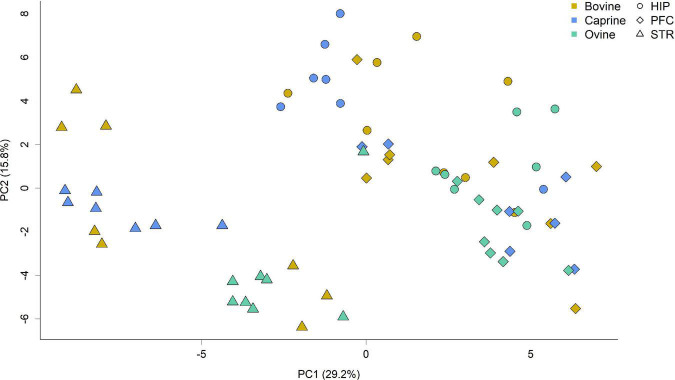
Principal component analysis score plot showing gene expression profiles of tissue samples from the hippocampus, striatum and prefrontal cortex of pigs fed milk from bovine, caprine, or ovine species. The first two principal components are plotted. Colors indicate milk treatments, and shape indicates different brain regions. Percentages of variation explained by each principal component are indicated along the axes. HIP, hippocampus; PFC, prefrontal cortex; STR, striatum; PC, principal component.

Gene expression patterns differed between the brain regions (Figure 1). The largest variation in gene expression profiles of brain tissue samples was seen in the principal component 1, with samples primarily grouped by brain region. Gene expression profiles in the striatum were distinct from the other areas, whereas the hippocampus and prefrontal cortex profiles overlapped to some extent. A secondary grouping of samples based on milk treatments was observed for the striatum for caprine and ovine milk-fed pigs, but this grouping was less evident for the hippocampus and prefrontal cortex.

### Differential gene expression

#### Brain region effect

Differences in gene expression levels between brain regions were observed, with 54 out of 77 genes being significantly different (FDR < 0.05; Table 3). Genes encoding for receptors for neurotransmitter glutamate (*GRID1*, *GRID2*, *GRM1*, *GRM7, GRM8* except for *GRM2*), serotonin (*HTR1B*, *HTR1D*, *HTR2C*, *HTR4* except for *HTR1E*), dopamine (*DRD1* and *DRD2*), neuropeptide Y (*NPY1R, NPY5R* except for *NPY2R*), and norepinephrine (*ADRA2B*, *ADRB1*, *ADRB2* except for *ADRA1D* and *ADRA2A*) had in general higher expression levels in the striatum than that of hippocampus and prefrontal cortex. Lower expression levels of genes encoding for receptors for neurotransmitter gamma-aminobutyric acid (*GABA*) (*GABRB2*, *GABRG2*, *GABRA1* except for *GABBR1*), histamine (*HRH1*), and acetylcholine (*CHRM3*) were observed in the striatum in comparison to the prefrontal cortex or hippocampus (Table 3).

**TABLE 3:** Expression levels of genes in the hippocampus (*n* = 22), prefrontal cortex (*n* = 23), and striatum (*n* = 23) tissue samples of pigs fed ovine, bovine, or caprine milk treatment*.

Category	Gene	Hippocampus	Prefrontal cortex	Striatum	FDR
Myelination marker	*CNP*	1305.22 ± 20.22^a^	1156.28 ± 18.42^b^	1272.62 ± 12.5^a^	2.63E-07
	*MAG*	1025.21 ± 21.66^a^	848.15 ± 24.28^b^	996.89 ± 16.09^a^	2.51E-07
	*MBP*	1426.16 ± 24.27^a^	1277.15 ± 24.31^b^	1375.92 ± 17.6^a^	9.44E-05
	*PLP1*	1360.7 ± 21.86^a^	1198.35 ± 17.97^b^	1326.39 ± 16.3^a^	2.38E-07
Neurotransmitter receptor	*ADRA1D*	397.24 ± 30.22^b^	719.69 ± 14.37^a^	312.71 ± 38.72^c^	1.01E-13
	*ADRA2A*	697.85 ± 21.56^a^	683.56 ± 9.71^a^	510.84 ± 23.64^b^	2.54E-09
	*ADRA2B*	471.87 ± 29.64^a^	305.36 ± 33.56^b^	544.93 ± 16.9^a^	3.44E-07
	*ADRB1*	633.38 ± 25.05^c^	693.28 ± 8.89^b^	791.14 ± 14.76^a^	2.02E-07
	*ADRB2*	521.43 ± 11.77^ab^	489.43 ± 22.71^b^	557.61 ± 11.89^a^	0.023704
	*CHRM3*	753.79 ± 8.06^c^	909.5 ± 5.73^a^	803.39 ± 12.45^b^	1.34E-16
	*DRD1*	677.83 ± 19.67^c^	816.52 ± 8.71^b^	1198.9 ± 16.27^a^	6.66E-32
	*DRD2*	461.92 ± 34.16^b^	374.39 ± 25.21^b^	1203.17 ± 34.25^a^	1.26E-27
	*GABBR1*	1264.34 ± 7.55^b^	1270.26 ± 7.29^b^	1364.38 ± 8.89^a^	3.47E-13
	*GABRA1*	1140.18 ± 17.52^b^	1255.49 ± 10.41^a^	1078.95 ± 12.53^c^	4.20E-12
	*GABRB2*	1092.72 ± 13.67^c^	1198.45 ± 7.53^a^	1151.92 ± 10.29^b^	3.38E-08
	*GABRG2*	1158.47 ± 9.32^a^	1164.38 ± 8.04^a^	1116.36 ± 6.89^b^	0.000204
	*GRID1*	862.76 ± 7.84^c^	891.95 ± 4.54^b^	986.23 ± 6.76^a^	3.51E-19
	*GRID2*	498.73 ± 49.82^b^	403.49 ± 49.75^b^	652.71 ± 50.06^a^	0.004225
	*GRM1*	752.41 ± 15.89^b^	617.43 ± 27.74^c^	811.72 ± 8.7^a^	7.52E-09
	*GRM2*	475.43 ± 34.2^b^	623.42 ± 21.61^a^	476.42 ± 31.25^b^	0.000903
	*GRM7*	889.54 ± 5.72^c^	919.08 ± 6.38^b^	967.55 ± 5.27^a^	1.70E-12
	*GRM8*	568.37 ± 27.88^c^	720.78 ± 12.26^b^	789.53 ± 14.25^a^	1.92E-10
	*HRH1*	704.37 ± 10.73^c^	862.75 ± 8.3^a^	741.52 ± 7.97^b^	1.00E-17
	*HTR1B*	383.98 ± 38.85^b^	380.44 ± 25.83^b^	741.42 ± 12.65^a^	2.98E-14
	*HTR1D*	434.39 ± 32.13^b^	392.05 ± 33.65^b^	791.13 ± 22.79^a^	7.62E-14
	*HTR1E*	626.42 ± 16.22^a^	579.41 ± 9.84^b^	560.02 ± 12.45^b^	0.003242
	*HTR2C*	360.44 ± 38.47^b^	268.26 ± 30.6^c^	717.08 ± 22.5^a^	1.58E-14
	*HTR4*	335.95 ± 27.06^b^	208.39 ± 22.93^c^	598.59 ± 15.53^a^	1.93E-17
	*NPY1R*	781.18 ± 13.49^b^	744.88 ± 8.92^c^	832.25 ± 6.84^a^	4.96E-07
	*NPY2R*	764.08 ± 26.8^a^	278.08 ± 30.99^c^	445.66 ± 20.37^b^	7.72E-18
	*NPY5R*	803.27 ± 11.28^b^	802.71 ± 5.1^b^	890.83 ± 7.03^a^	2.17E-11
Neurotransmitter transporter	*SLC1A2*	1145.76 ± 8.48^c^	1169.84 ± 5.78^b^	1213.53 ± 7.12^a^	7.45E-08
	*SLC1A3*	1126.62 ± 6.19^c^	1171.01 ± 5.99^b^	1212.58 ± 7.96^a^	1.94E-11
	*SLC5A7*	462.45 ± 22.56^b^	372.9 ± 23.81^c^	813.26 ± 28.98^a^	8.67E-18
	*SLC6A1*	1060.1 ± 17.42^b^	1066.84 ± 14.9^b^	1133.21 ± 15.56^a^	0.004225
	*SLC6A11*	873.74 ± 14.71^b^	898.82 ± 8.32^ab^	921.46 ± 12.36^a^	0.035159
Neurotransmitter enzyme	*ACHE*	913.28 ± 12.65^b^	902.31 ± 8.99^b^	1000.04 ± 9.3^a^	8.53E-09
	*DBH*	142.8 ± 24.9^b^	231.02 ± 29.31^a^	232.38 ± 24.23^a^	0.040051
	*DDC*	546 ± 30.5^a^	438.73 ± 25.96^b^	615.04 ± 21.11^a^	8.14E-05
	*GAD1*	1120.74 ± 9.56^b^	1144.54 ± 6.56^b^	1230.48 ± 9.09^a^	1.33E-12
	*GAD2*	1072.19 ± 11.43^b^	1098.65 ± 8.92^b^	1249.28 ± 13.82^a^	8.01E-16
Neurotrophin	*BDNF*	824.76 ± 15.71^a^	794.94 ± 9.34^a^	401.08 ± 30.83^b^	5.28E-22
	*NTF3*	215 ± 30.78^a^	253.59 ± 26.55^a^	91.13 ± 22.52^b^	0.000253
	*NTRK3*	776.89 ± 18.67^a^	682.95 ± 24.05^b^	825.92 ± 27.37^a^	0.000422
Synaptic vesicle cycle	*CPLX4*	292.47 ± 23.42^a^	211.17 ± 21.21^b^	308.37 ± 24.01^a^	0.011492
	*RIMS1*	1079.98 ± 9.96^b^	1138.92 ± 6.79^a^	1057.74 ± 6.38^c^	2.70E-09
	*SNAP25*	1362.91 ± 5.13^c^	1402.06 ± 6.05^a^	1380.86 ± 5.16^b^	4.40E-05
	*STXBP1*	1160.96 ± 7.83^b^	1208.42 ± 7.97^a^	1188.04 ± 9.67^a^	0.001635
	*SYT1*	1178.5 ± 13.27^a^	1191.9 ± 14.02^a^	1091.12 ± 23.84^b^	0.000416
Synaptogenesis marker	*DLG4*	1177 ± 7^c^	1217.53 ± 7.09^b^	1249.69 ± 6.62^a^	6.29E-09
	*GAP43*	1184.14 ± 20.49^b^	1258.28 ± 13.62^a^	1277.53 ± 17.1*^a^*	0.001178
	*SYN1*	1218.65 ± 4.69^b^	1259.07 ± 6.04^a^	1193.45 ± 8.45^c^	2.00E-08
	*SYN2*	1077.62 ± 10.44^a^	1069.04 ± 8.46^a^	1020.33 ± 10.72^b^	0.000353
	*SYN3*	813.8 ± 15.38^b^	814.16 ± 6.88^b^	937.92 ± 7.19^a^	8.80E-13

*Only genes whose expressions were significantly different between brain tissue types are shown. Data were analyzed via one-way ANOVA with post hoc Fisher’s Least Significant Difference.

^a–c^Values with different superscript letters in the same row differ (FDR < 0.05). Values are represented as mean ± standard error of the mean.

FDR, false discovery rate; n, number of samples.

The genes encoding for enzymes involved in the synthesis of the neurotransmitter acetylcholine (*ACHE*), dopamine (*DDC*), glutamate (*GAD1* and *GAD2*), norepinephrine (*DBH*), and transporters of neurotransmitter glutamate (*SLC1A2* and *SLC1A3*), acetylcholine (*SLC5A7*), and GABA (*SLC6A1* and *SLC6A11*) had in general higher expression levels in the striatum than those of the hippocampus and prefrontal cortex. In addition, myelination marker (*MAG*, *MBP*, *PLP1*, and *CNP*) gene expression levels were higher in the striatum and hippocampus compared to the prefrontal cortex.

The mRNA abundance of genes encoding for synaptogenesis (*DLG4*, *GAP43*, and *SYN3*) was higher in the striatum than in the prefrontal cortex and hippocampus. In contrast, synapsin isomer (*SYN1* and *SYN2*) gene expression levels were either higher in the prefrontal cortex or similar to the hippocampus compared to the striatum. In addition, the expression levels of neurotrophin genes (*BDNF* and *NTF3*) and synaptic vesicle cycle genes (*SNAP25*, *STXBP1*, *SYT1*, and *RIMS1*) except for *CPLX4* were, in general, higher in the prefrontal cortex than in striatum and hippocampus. Overall, genes in the striatum showed increased expression compared to the hippocampus and prefrontal cortex (Supplementary Figure 3).

#### Milk treatment effect

**FIGURE 2:**
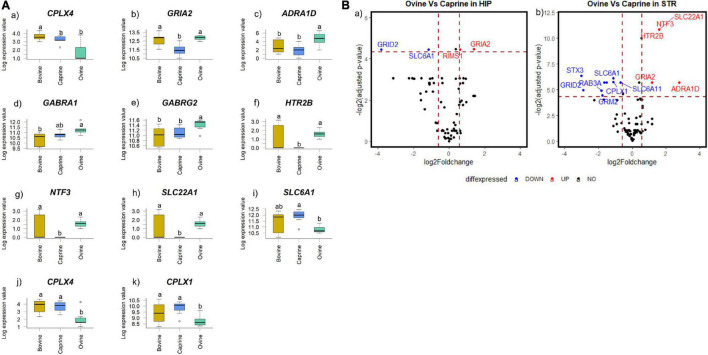
**(A)** Boxplot showing the genes with significant changes in expression levels in **(a,b)** hippocampal and **(c–k)** striatal tissues of pigs fed milk from bovine, caprine or ovine species. The black line in each box indicates the median value, the height of the box represents the interquartile range, and the whiskers of each box indicate the most extreme values within 1.5 times of the interquartile range. Outliers are shown as individual circles. Milk treatments with different letters differ significantly (FDR < 0.05 for hippocampal genes and FDR < 0.1 for striatal genes). **(B)** Volcano plot showing a pairwise comparison of the differential expression of genes between milk treatment groups **(a)** ovine versus caprine in the hippocampus and **(b)** ovine versus caprine in the striatum. The dotted vertical line indicates fold change value ± 1.5, and the dotted horizontal lines indicate FDR adjusted *P*-value < 0.05 threshold. Scattered points represent genes: black points indicate genes that are not differentially expressed, blue points indicate genes that are significantly lower in expression, and red points indicate genes that are significantly higher in expression. Only statistically significant genes (identified using a *t*-test) are labeled. HIP, hippocampus; STR, striatum; FDR, false discovery rate.

Ruminant milk treatment affected the brain gene expression profile of pigs in a brain region-dependent manner. *CPLX4* expression was affected by milk treatments both in the hippocampus and striatum (Figures 2Aa,j). In the hippocampus, *GRIA2* expression was significantly different in response to milk treatments (FDR < 0.05; Figure 2Ab). In the striatum, *ADRA1D*, *CPLX1*, *GABRA1*, *GABRG2, HTR2B*, *NTF3*, *SLC22A1*, and *SLC6A1* expression levels were significantly different in response to milk treatments (FDR < 0.1; Figure 2A). No genes in the prefrontal cortex significantly differed in expression in response to milk treatments (FDR > 0.1) (data not shown). *HTR2B, NTF3*, and *SLC22A1* expressions were not detected in the striatum tissue samples of pigs fed with caprine milk (Figure 2A).

The pairwise differential gene expression between milk treatment groups was visualized using a volcano plot (Figure 2B). Significant gene expression FC (FC > 1.5 and FDR < 0.05) in the hippocampus and striatum were identified only between the ovine and caprine groups. No significant gene expression FC (FC > 1.5 and/or FDR > 0.05) was identified between ovine vs bovine and caprine vs bovine milk groups (data not shown). In the hippocampus, the expression levels of two genes (*SLC6A1* and *GRID2*) were decreased by >1.5-fold in the ovine milk group, while *GRIA2* and *RIMS1* increased by >1.5-fold in the ovine milk group compared to the caprine milk group. The expression levels of seven genes (*CPLX1, GRID2, GRM2, RAB3A, STX3, SLC6A1*, and *SLC6A11*) in the striatum were decreased by >1.5-fold in the ovine milk group compared to the caprine milk-fed pigs. On the other hand, the expression levels of five genes (*ADRA1D*, *GRIA2*, *HTR2B*, *NTF3*, and *SLC22A1*) in the ovine milk group were increased by >1.5-fold compared to the caprine milk group.

### Milk nutrient intake and brain gene expression correlations

In this study, pigs received different volumes of milk to match the protein intake between milk groups from PND14 or 15, resulting in different amounts of milk nutrients being fed to the pigs (Table 2). Hence, a correlation analysis was performed to identify whether the amount of milk nutrient intake influenced brain gene expression.

**FIGURE 3:**
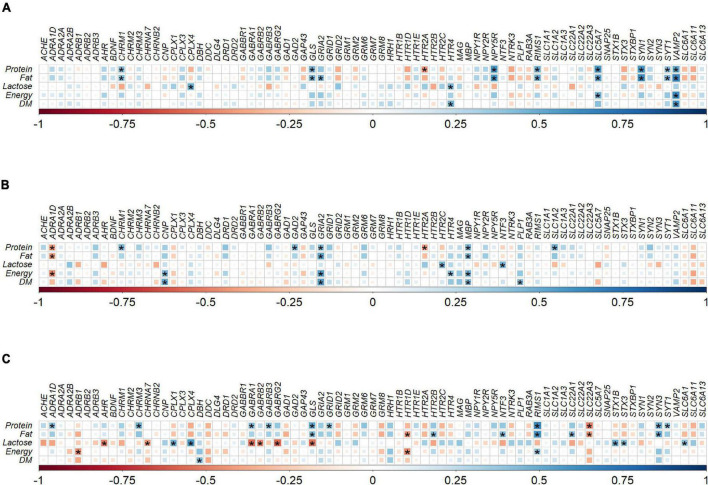
Correlation plot depiction of the Spearman correlations between milk nutrient intake and gene expression levels in **(A)** hippocampus (*n* = 22), **(B)** prefrontal cortex (*n* = 23), and **(C)** striatum (*n* = 23) tissue samples obtained from pigs fed with milk from bovine, ovine or caprine species. The color and size of the squares indicate the magnitude of the correlation, i.e., blue indicates positive correlation and red indicates negative correlation. Asterisks indicate the significance of correlation (*P* < 0.05). The legend at the bottom of each correlation plot shows the correlation coefficients with their corresponding colors. DM, dry matter; *n*, number of samples used for correlation.

Spearman correlation analysis identified potential relationships between protein, fat, lactose, energy, and dry matter intakes of the milk (from PND 9 or 10 onward) from different ruminant species and the expression levels of the 77 genes of the individual brain regions (Figure 3).

In the hippocampus there were a total of 23 positive and one negative correlations between milk nutrient intakes and gene expression levels. However, significant positive correlations (*P* < 0.05, rho > ±0.5) were identified between *NPY5R* and fat intake (rho = 0.57), *SLC5A7* and protein intake (rho = 0.58), *SLC5A7* and fat intake (rho = 0.51), *VAMP2* and protein intake (rho = 0.52), *VAMP2* and fat intake (rho = 0.65), *VAMP2* and energy intake (rho = 0.62), and *VAMP2* and dry matter intake (rho = 0.57). No significant negative correlations (*P* > 0.05 or rho < ±0.5) were found in hippocampal gene expression and milk nutrients (Figure 3A).

In the prefrontal cortex, there were 17 positive and four negative correlations between milk nutrient intakes and gene expression levels. Significant positive correlation was identified between *GRIA2* and fat intake (rho = 0.51), *GRIA2* and energy intake (rho = 0.54), and *GRIA2* and dry matter intake (rho = 0.54). No significant negative correlations (*P* > 0.05 or rho < ±0.5) were found between prefrontal cortex gene expression and milk nutrients (Figure 3B).

In the striatum, there were 22 positive and 11 negative correlations between milk nutrient intakes and gene expression levels. Significant positive correlation was identified between *CPLX4* and lactose intake (rho = 0.57), GLS and protein intake (rho = 0.51), *RIMS1* and protein intake (rho = 0.62), and *RIMS1* and fat intake (rho = 0.59). Significant negative correlations were identified in the striatum between *GABRA1* and lactose intake (rho = −0.54), *GABRG2* and lactose intake (rho = −0.57), *GLS* and lactose intake (rho = −0.53), and *SLC22A3* and protein intake (rho = −0.50; Figure 3C).

## Discussion

This study is the first to show the effects of feeding whole milk from different ruminant species on the mRNA expression of genes in brain regions associated with cognitive function in pigs at approximately PND 21, a well-recognized model of human infant brain development (Guilloteau et al., 2010; Mudd and Dilger, 2017). The results outlined in this study demonstrated that different milk treatments influenced the mRNA expression of specific genes encoding for cognitive functions within the brain, and some of these gene expression changes were associated with nutrient intake.

### Brain-region gene expression differences

Differences in gene expression were observed between brain regions, regardless of the milk treatments. These differences most likely reflect the developmental heterogeneity and functional differences between brain regions (Knickmeyer et al., 2008; Jena et al., 2020). In this study, genes encoding neurotransmitter receptors, enzymes, and transporters in the striatum had increased expression levels compared to the hippocampus and prefrontal cortex, suggesting more neurotransmission in the striatum to serve its functional needs. Similarly, in the prefrontal cortex, the genes associated with myelination showed decreased expression levels, which could be explained by the late development of the prefrontal cortex compared to the other regions (Huttenlocher and Dabholkar, 1997; Barkovich, 2005).

### Brain gene expression differences between different milk

Milk effects on gene expression were brain region-specific. Hippocampal and striatal gene expression profiles were affected by milk treatments, whereas no effect was observed in the prefrontal cortex. One reason for such effect may be differences in the rate of myelination for different brain areas. Myelination progresses from caudal to cephalad, i.e., subcortical brain areas (hippocampus, striatum) would be myelinated before cortical area (e.g., prefrontal cortex) (Barkovich, 2005). Interestingly, the myelin sheath is mainly composed of lipids (Barkovich, 2005), and studies with human infants and rodent models have shown that dietary lipids (e.g., sphingomyelin and fatty acids) are positively associated with myelination (Salvati et al., 1996; Deoni et al., 2018). As lipids are an important source of nutrients in milk (Koletzko, 2017), regardless of the species, it is plausible that lipids can influence myelination, but how remains unknown. However, as the prefrontal cortex is the last to get myelinated compared to the hippocampus and striatum (Volpe, 2000; Barkovich, 2005), it can be speculated that the requirement of lipids for myelin synthesis in the prefrontal cortex would not be as necessary during the initial 3 months of life as for other brain regions, hence the effect.

In the hippocampus and striatum, genes related to synaptic vesicle cycle, glutamatergic, GABAergic, serotonergic, and adrenergic transmission, and neurotrophin signaling were most affected by the ruminant milk treatments. Other studies have shown that lower gene expression levels in the hippocampus and striatum associated with these processes were implicated in behavior changes involving memory impairment in rodent models (Mathew et al., 2010; Tellez et al., 2012; Xie et al., 2015; Ramos-Miguel et al., 2017; Korz et al., 2021). However, the present study did not ascertain any functional relevance of these changes, i.e., whether the level of changes in gene expression between milk treatments was sufficient to change brain function.

Notable differences in gene expression levels were only observed between ovine and caprine milk treatments. In contrast, gene expression levels between bovine and caprine or bovine and ovine milk treatments were similar. These differences in gene expression patterns could be attributed to differences in nutrient concentration between ruminant milk types. Ovine milk has a distinct nutrient profile with a higher protein, fat, energy and dry matter content than caprine and bovine milk, as shown in Supplementary Table 1 and other studies (Barlowska et al., 2011; Claeys et al., 2014). However, in this study, pigs received a milk diet balanced for milk protein content from PND 14 or 15, changing the volume of each milk fed to pigs, resulting in varying amounts of other milk nutrients consumed (Table 2). For instance, pigs in the ovine milk group received more fats than other milk groups, considering the per day average nutrient intake of the entire study period. It is noteworthy that even if the protein contents were matched between the milk groups, pigs received a different amount of proteins in their milk diets per day on average (Table 2) because milk nutrient intake was calculated considering the whole study period (except the first 3 days), which included the days when milk was not balanced for protein content (before PND 14 or 15). Hence, based on the study design, the observed differences in the gene expression levels between ovine and caprine milk groups could not be ascribed to the nutrient concentration difference in the original milk. Instead, the effects could be attributed to the amount of each nutrient in the milk intake of pigs.

Differences in composition of lipids (Park et al., 2007; Felice et al., 2021), amino acids (Claeys et al., 2014; Rafiq et al., 2016), and oligosaccharides (Van Leeuwen et al., 2020; Shi et al., 2021) also exist between ruminant milk types. For instance, ovine milk has higher tryptophan and glutamate than caprine milk, as reported in other studies (Claeys et al., 2014; Rafiq et al., 2016). Additionally, ovine milk has higher unsaturated fatty acid levels and lower saturated fatty acid levels than caprine milk (Park et al., 2007; Felice et al., 2021). Given the higher amounts of protein and fat in the intake of the ovine milk in this study, it is likely that specific macronutrient constituents (e.g., tryptophan, glutamate, unsaturated fatty acids) would also have been fed in a higher amount to the pigs of the ovine milk group. Although the concentration of amino acids and fatty acids in the milk has not been evaluated, these macronutrient constituents have the potential to alter brain gene expression.

Dietary amino acids are absorbed and metabolized in the gut mucosa and liver and subsequently released in the systemic circulation, where selected amino acids (e.g., tryptophan, phenylalanine) can cross the blood-brain barrier and act as precursors for the synthesis of neurotransmitters in the brain (Zaragozá, 2020). Amino acids can also be precursors of various neurotransmitters produced by the gut microbiota and influence brain function *via* mediators of the gut-brain axis (Jena et al., 2020). So, it is plausible that the pigs in the ovine milk group would have increased mRNA abundance of the receptor for specific neurotransmitters in the brain to facilitate binding of the neurotransmitter produced through amino acid metabolism. For instance, for serotonin (*HTR2B*) and glutamate (*GRIA2*) receptor genes, ovine milk-fed pigs showed increased expression levels in the hippocampus and striatum compared to the caprine milk group, suggesting these effects could be a result of differences in amino acid intake between milk treatments.

A study had shown that feeding diets with high saturated fat before and during early postnatal periods was associated with decreased expression of hippocampal neurotrophin (*BDNF*), genes encoding for synaptic vesicle proteins (*SYN*), and glutamatergic receptor (*GRIN2B*), as well as learning and memory deficits in the rats (Page and Anday, 2020). Pigs fed the ovine milk would have consumed higher amounts of total fat and lower amounts of saturated fat, agreeing with results reported in other studies (Park et al., 2007; Felice et al., 2021). Therefore, it would be expected that the genes associated with these processes would have higher expression in the ovine milk group. However, in the current study, genes associated with similar processes were either higher (*NTF3*, *GRIA2*) or lower (*CPLX4*, *CPLX1*) in the ovine milk group than in the caprine milk group. This finding suggests that the milk lipid constituents may regulate different genes differently. Surprisingly, milk treatments did not affect myelination-associated genes in any brain area in this study, even if studies have shown that lipids affect myelination (as described above). This effect could be due to insufficient differences in intake of specific lipid constituents between milk treatments or a consequence of the short feeding period to change myelination-associated gene expression.

### Milk nutrient intake association with brain gene expression

Milk lactose was positively or negatively correlated with the striatal *CPLX4*, *GABRA1*, and *GABRG2* mRNA counts; these gene expression levels differed between milk treatments. This finding suggests a possible role of lactose intake on specific brain gene expression, yet unknown. This correlation could be explained by the absorption of lactose and indirectly through microbial lactose fermentation. Lactose in the small intestine is broken down to glucose and galactose and subsequently absorbed into the bloodstream for energy. A study has shown that rats can metabolize glucose and galactose to amino acids (glutamate, glutamine, and GABA) in the brain (Roser et al., 2009). Thus, it is likely that the change in amino acid (or neurotransmitter) levels in the brain might have influenced its receptor expression. Another study involving *in vitro* fermentation of skimmed milk showed that *Levilactobacillus brevis* co-cultured with *Streptococcus thermophilus* produces GABA neurotransmitters by utilizing lactose as the primary source of carbon (Xiao et al., 2020), albeit a limited amount of lactose should be available for large intestinal fermentation *in vivo*. Other studies suggest that circulating GABA can cross the blood-brain barrier to influence brain function (Al-Sarraf, 2002; Shyamaladevi et al., 2002).

### Strengths and limitations

The application of the Nanostring technique for analyzing the brain gene expression is one of the main strengths of this study. This method detects the true population of mRNA in the samples and offers an amplification free detection of mRNA (Wang et al., 2016). Other methods, like RNA sequencing, require enzymatic reactions like reverse transcription and polymerization, making the measurement indirect; it also does not provide accurate mRNA quantification (Conesa et al., 2016).

The pigs in this study received the diet on a per kg BW basis, and these differences in milk intake may have resulted in some differences in the brain gene expression as heavier animals received more milk than lighter animals. It is also important to note that other nutrients in milk (e.g., oligosaccharides, fatty acids) than those analyzed could have explained associations between the milk treatments and brain gene expression changes. Unlike some animal studies, this study removed the confounding effect of uncontrolled food intake by fasting the pigs and euthanizing them at the same post-feeding time, making this a more controlled study.

Another limitation is that the gene expression responses were only studied in male pigs. However, a study has shown that the expression of *BDNF* in the hippocampus in germ-free mice was sex-dependent (Clarke et al., 2013). Hence, including female pigs in the study would have been informative, but it will have required more pigs per treatment beyond the scope of this study.

It is acknowledged that using a reference group, i.e., pigs fed with mother’s milk showing basal expected brain gene expression would have been relevant to compare with the pigs fed with other species’ milk. However, the brain samples used were collected from a study that aimed to compare structural changes in bovine with non-bovine (ovine and caprine) whole milk on digestion of pigs in early postnatal life, and using pigs fed with mother’s milk was not required for the experiment. Future studies should compare where possible mRNA gene expression levels of pigs fed with mother milk and those fed with milk from other species.

Furthermore, changes in gene expression do not necessarily translate to changes in protein expression or physiological function. However, they are the essential first step of most biological processes. Therefore, future studies should measure changes in the abundance of proteins involved in cognitive function and associated behaviors. These data would help understand whether the observed gene expression changes in response to the consumption of milk from bovine, caprine and ovine milk would contribute to changes in brain function and behavior in early postnatal life.

## Conclusion

This study is the first to investigate the effects of consuming whole milk from different ruminant species on expression of genes related to cognitive function in the brain in the early postnatal life of pigs as a model for human infants. It was shown that different ruminant milk treatments, consumed at different volumes to balance protein intake, altered the expression of neurotransmission genes that are important for cognitive development in early postnatal life. This study also highlighted a brain region-specific milk effect, suggesting the importance of studying individual brain regions rather than the whole brain as one homogenous organ. While ruminant milk is not an alternative to human milk, this study provides novel insights on the biological impact of whole milk from different ruminant species, which can be applied to design nutritionally advanced infant formulas.

## Data availability statement

The NanoString data has been deposited in the NCBI GEO under accession number GSE199988. All data generated or analyzed during this study are included in this article/Supplementary material.

## Ethics statement

The animal study was reviewed and approved by Massey University, Animal Ethics Committee (MUAEC protocol 18/97).

## Author contributions

DR, CM, and WM co-designed the animal experiment. DR and CM conducted the pig experiment. AJ assisted with collecting biological samples, conducted the laboratory experiments and data analysis, and wrote the original draft. CM and NR helped in structuring the manuscript and critically reviewed the manuscript. WY and CM helped with the statistical analyses. JM helped in the selection of the gene panel for NanoString. CM, NR, WY, JM, WM, RD, and CG advised and critically reviewed the manuscript. WM and NR sourced the funding for the study. All authors contributed to the article and approved the submitted version.

## Abbreviations

PND: postnatal day
FDR: false discovery rate
FC: fold change
BW: body weight
BDNF: brain-derived neurotrophic factor.
