# Supplementary material for: The effects of ruminant milk treatments on hippocampal, striatal, and prefrontal cortex gene expression in pigs as a model for the human infant
Source: Front Neurosci. 2022 Aug 15;16:937845. doi: 10.3389/fnins.2022.937845 (PMC9421158; doi:10.3389/fnins.2022.937845)
Supplement: Supplementary file 1 [file Data_Sheet_1.docx]

Supplementary Material

# Supplementary Data

## Supplementary Table

**Table S1** Chemical composition (g/100g) of bovine, caprine and ovine whole milk*

| **Component** | **Bovine** | **Caprine** | **Ovine** |
| --- | --- | --- | --- |
| Dry matter | 13.1 ± 0.11^b^ | 11.23 ± 0.17^c^ | 17.6 ± 0.05^a^ |
| Protein | 3.61 ± 0.04^b^ | 3.16 ± 0.06^c^ | 6.27 ± 0.04^a^ |
| Fat | 4.06 ± 0.15^b^ | 3.22 ± 0.1^c^ | 6.31 ± 0.05^a^ |
| Lactose | 4.56 ± 0.05^a^ | 3.91 ± 0.04^c^ | 4.16 ± 0.03^b^ |
| Gross energy | 75.80 ± 1.05^b^ | 61.11 ± 0.7^c^ | 107.50 ± 0.15^a^ |

^a-c^Values with different superscript letters in the same row differ significantly (P < 0.05). Data were analysed via one-way analysis of variance with post-hoc Fisher’s Least Significant Difference. *Values are represented as mean ± standard error of the mean of three different batches of milk.

## Supplementary Figure


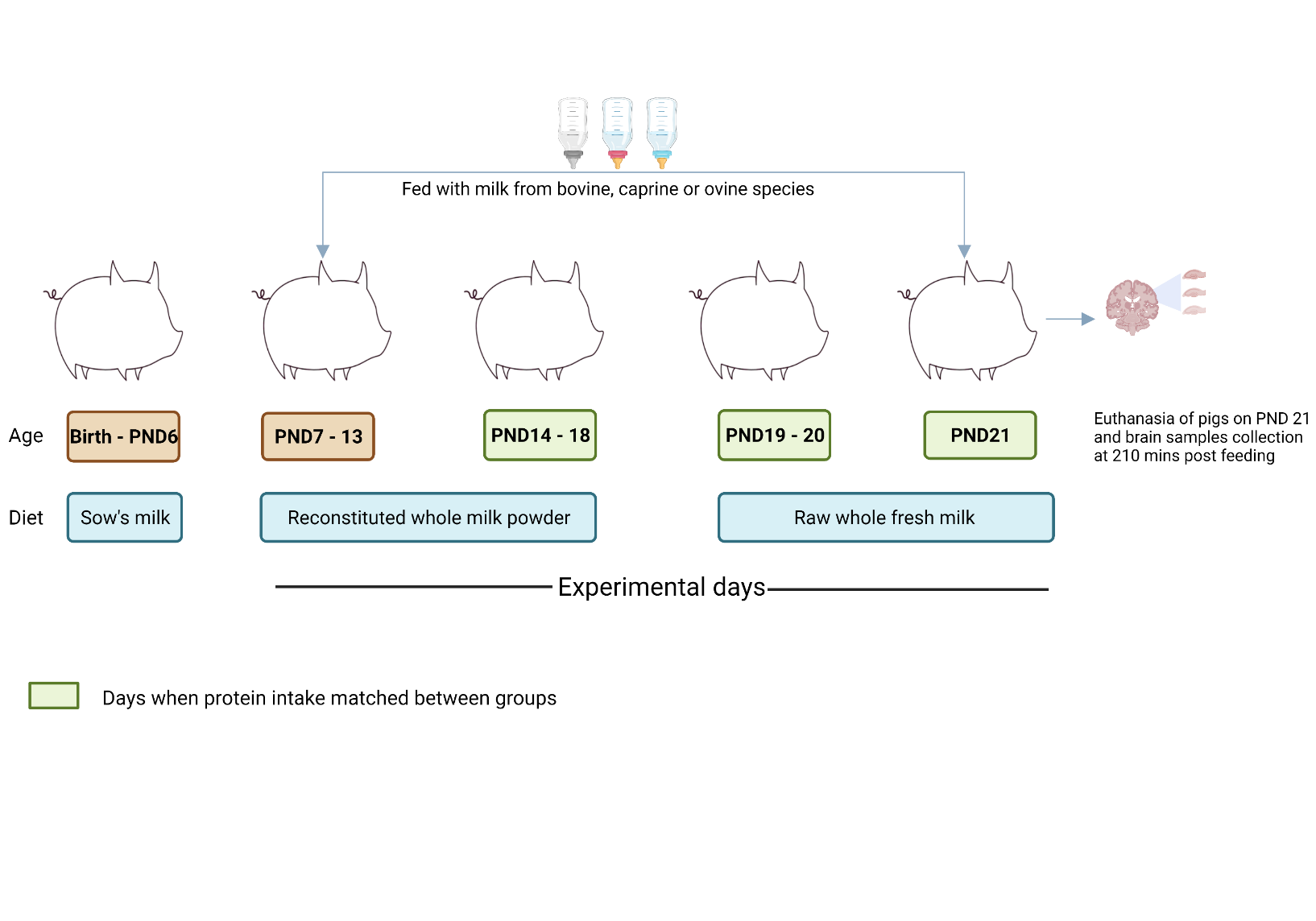


**Figure S1** Overview of the *in vivo* pig study. PND, postnatal day. Figure created with biorender.com

**
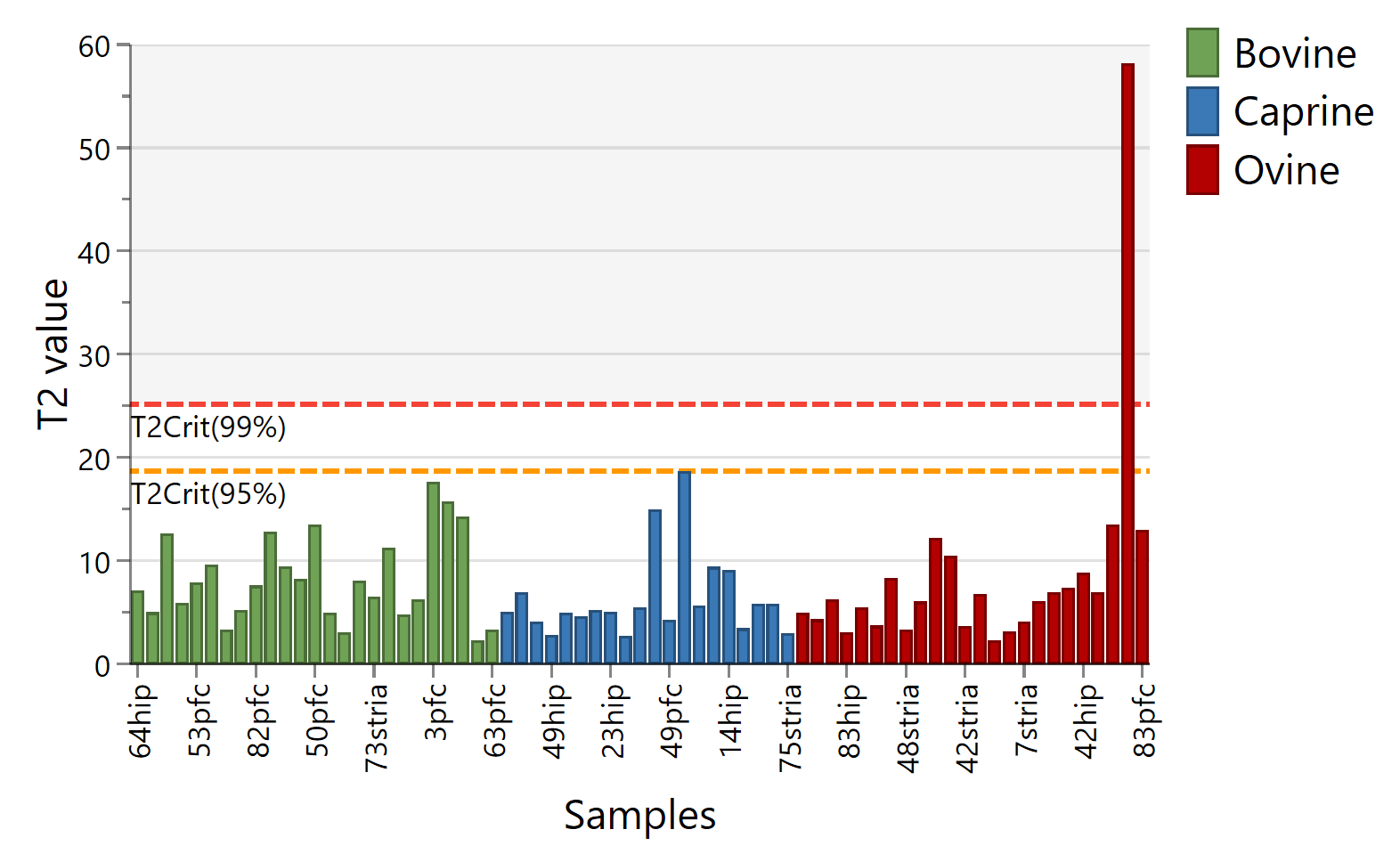
**

**Figure S2** Hotelling T2 plot showing potential outlier among the brain tissue samples of the pigs exclusively fed with bovine, caprine or ovine milk for 15 days. The red and orange dotted line represent the 99% and 95% confidence intervals. A T2 value larger than the 99% confidence indicates that the sample is far away from other samples, and the probability of it belonging to the same class as other samples is lower than 1 %, hence an outlier.


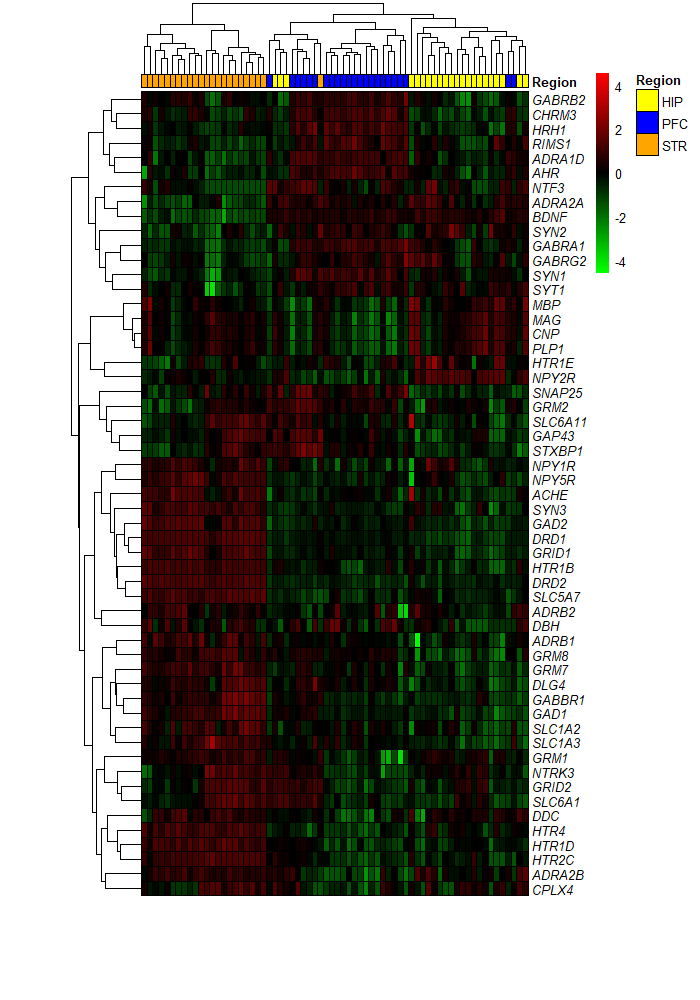


**Figure S3** Heatmap showing hierarchical clustering of significantly expressed genes between the hippocampus, prefrontal cortex, and striatum tissue samples of piglets fed milk from ovine, bovine, or caprine species. Heatmap colour indicates normalised (Z score) mRNA counts scaled across rows. The intensity of red colour denotes the number of standard deviations above the mean (increased gene expression levels) and intensity of green colour denotes the number of standard deviations below the mean (decreased gene expression levels). HIP, hippocampus; PFC, prefrontal cortex; STR, striatum.
